# The *Mycobacterium tuberculosis* MmpL3 inhibitor MSU-43085 is active in a mouse model of infection

**DOI:** 10.1128/spectrum.03677-23

**Published:** 2023-12-11

**Authors:** John T. Williams, Matthew Giletto, Elizabeth R. Haiderer, Bilal Aleiwi, Teresa Krieger-Burke, Edmund Ellsworth, Robert B. Abramovitch

**Affiliations:** 1 Department of Microbiology and Molecular Genetics, Michigan State University, East Lansing, Michigan, USA; 2 Department of Pharmacology and Toxicology, Michigan State University, East Lansing, Michigan, USA; Johns Hopkins University School of Medicine, Baltimore, Maryland, USA

**Keywords:** *Mycobacterium tuberculosis*, nontuberculous mycobacteria, drug development, MmpL3

## Abstract

Addressing drug resistance in *Mycobacterium tuberculosis* (Mtb) requires the development of new drugs with new targets. We previously identified 13 MmpL3 inhibitors from a screen of a small library of Mtb growth inhibitors. In this report, we describe the biological activities of a set of HC2099 analogs active against Mtb *in vitro*, intracellular Mtb, as well as *M. abscessus* and *M. avium*. Pharmacokinetic (PK) studies identified MSU-43085 as orally bioavailable with a short half-life. High dosing (100 mg/kg) saturates clearance processes, enabling efficacy studies. The results of an *in vivo* efficacy study found that MSU-43085 was active against Mtb in an acute murine TB infection model but lacked activity in a chronic murine TB infection model. The results of this study serve as a proof of concept for this series and support further optimizations of the HC2099 series of MmpL3 inhibitors.

**IMPORTANCE:**

MmpL3 is a protein that is required for the survival of bacteria that cause tuberculosis (TB) and nontuberculous mycobacterial (NTM) infections. This report describes the discovery and characterization of a new small molecule, MSU-43085, that targets MmpL3 and is a potent inhibitor of *Mycobacterium tuberculosis* (Mtb) and *M. abscessus* survival. MSU-43085 is shown to be orally bioavailable and efficacious in an acute model of Mtb infection. However, the analog is inactive against Mtb in chronically infected mice. Pharmacokinetic and metabolite identification studies identified *in vivo* metabolism of MSU-43085, leading to a short half-life in treated mice. These proof-of-concept studies will guide further development of the MSU-43085 series for the treatment of TB or NTM infections.

## INTRODUCTION

*Mycobacterium tuberculosis* (Mtb) is the primary causative agent of tuberculosis (TB). Current TB standard-of-care requires a 6-month regimen of four drugs: rifampin (RIF), isoniazid (INH), ethambutol (EMB), and pyrazinamide. The evolution of multi-drug resistance (MDR) and extensively drug-resistant strains of Mtb, which spread person-to-person (1), requires the development of new therapeutic strategies with novel mechanisms of action (MOA). The essential mycolic acid flippase (2) MmpL3 has been identified as a new potential target for TB therapy (3 – 20) with more than 20 different chemical scaffolds reported (21). SQ109, the most clinically advanced MmpL3 inhibitor, completed a Phase IIb clinical trial in 2018 (22). Although SQ109 has favorable features, including actively killing both replicating and non-replicating Mtb *in vitro* (20, 23), it has a short *in vivo* half-life owing to degradation by cytochrome P450 (CYP) enzymes CYPD6 and CYP2C19 (24 – 26). Additionally, SQ109 is not effective against non-tuberculosis mycobacteria (NTM), such as *M. abscessus* (MAB) or *M. avium* complex (MAC) (27). These observations necessitate the development of additional MmpL3 inhibitors for clinical applications, a conserved target across the in several mycobacterial species.

We have previously reported a targeted mutant screening approach for the identification of novel MmpL3 inhibitors (18). This screen identified 13 MmpL3 series, including the previously identified SQ109 (16), C215 (15), and HC2091 (20), that inhibit Mtb both *in vitro* and in infected macrophages. Two structurally similar compounds were identified, HC2099 and HC2183, with both having modest activities against Mtb *in vitro* [half-maximal effective concentrations (EC_50_s) of 1.7 µM and 3.2 µM, respectively] and no detectable cytotoxicity in macrophages. They demonstrated promising activities against intracellular Mtb (EC_50_ of <0.3 µM and 3.0 µM for HC2099 and HC2183, respectively), with HC2099 demonstrating a fivefold enhanced intra-macrophage activity compared to *in vitro* activity. These compounds also exhibit favorable ADME properties, including high solubility and microsome stability (18), making them candidates for structure-activity relationship (SAR) studies.

Other MmpL3 inhibitors, sharing some structural features relative to HC2099, have been described in the literature (28). These include the 1H-benzimidazole MmpL3 inhibitors EJMCh4 and EJMCh6, which are potent inhibitors against both Mtb and NTMs (8, 29). This series of MmpL3 inhibitors was active against intracellular Mtb in infected macrophages (8) and demonstrated efficacy in a *M. abscessus* zebrafish infection model (29). Similarly, the NITD series, exemplified by NITD-304 and NITD-349, are highly potent MmpL3 inhibitors with MICs reported as 20 and 30 nM, respectively (12). These analogs have activities against Mtb in both acute and chronic murine infection models (12). Based on the similarity between these compounds and parent compound HC2099, we sought to further optimize HC2099 through SAR studies to identify analogs demonstrating *in vivo* efficacy.

Using a mixed *mmpL3* mutant population and an *mmpR5* efflux strain, we demonstrate that active compounds target MmpL3 and are not susceptible to MmpR5-regulated efflux. Three of these compounds, MSU-43085, MSU-43165, and MSU-43170, demonstrate drug-like properties including favorable solubility, microsomal stability, and low cytotoxicity. MSU-43085 demonstrates activity against MAB and MAC *in vitro* and in macrophages, with activity comparable to the standard-of-care drugs amikacin and clarithromycin. *In vivo* studies show that these analogs demonstrate oral bioavailability and are well tolerated in mice treated at a high dose. Using an acute infection model, we demonstrated that MSU-43085 prevented Mtb growth in C57Bl/6 mice in an acute infection model. However, it was not effective in a study using the chronic TB infection model. These results provide proof of concept for a new, orally bioavailable series of MmpL3 inhibitors and support further pre-clinical studies.

## RESULTS

### HC2099 analogs are potent Mtb inhibitors

In a previous report, we identified 13 MmpL3 inhibitors using a combined untargeted and targeted whole-cell phenotypic mutant screen (18). Of these 13 compounds, 3 (SQ109, C215, and HC2091) were previously identified as MmpL3 inhibitors (15, 16, 20), while the remaining 10 (HC2032, HC2060, HC2099, HC2134, HC2138, HC2149, HC2169, HC2178, and HC2184) were novel or new versions of previously described chemical scaffolds (18). HC2099 and HC2183 are structural analogs, differing by a single substitution on the benzimidazole ring (-Cl vs -CH_3_ substitution) (Table 1). These two compounds were active against Mtb both *in vitro* and intra-macrophage and had favorable kinetic solubility and microsome stability (18).

**TABLE 1:** *In vitro* and cytotoxicity activities of HC2099 analogs

ID	MW (g/mol)	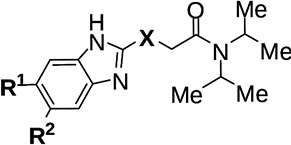			*In vitro* EC_50_ (μM)	*In vitro* EC_90_ (μM)	CC_50_ (μM)	Si (CC_50_/EC_50_)
		X	R^1^	R^2^				
HC2099	325.1	-S-	Cl	H	5.9	13.7	>80	>13
HC2183	305.2	-S-	Me	H	7.3	16.0	>80	>11
MSU-42766	319.2	-S-	Me	Me	1.3	8.2	>80	>61
MSU-43065	301.4	-CH_2_-	Me	Me	0.46	0.77	>80	>173
MSU-43085	342.3	-CH_2_-	Cl	Cl	0.12	0.25	>80	>667
MSU-43165	342.1	-NH-	Cl	Cl	0.38	0.63	>80	>210
MSU-43170	343.1	-O-	Cl	Cl	0.12	0.26	>80	>667

**Fig 1:**
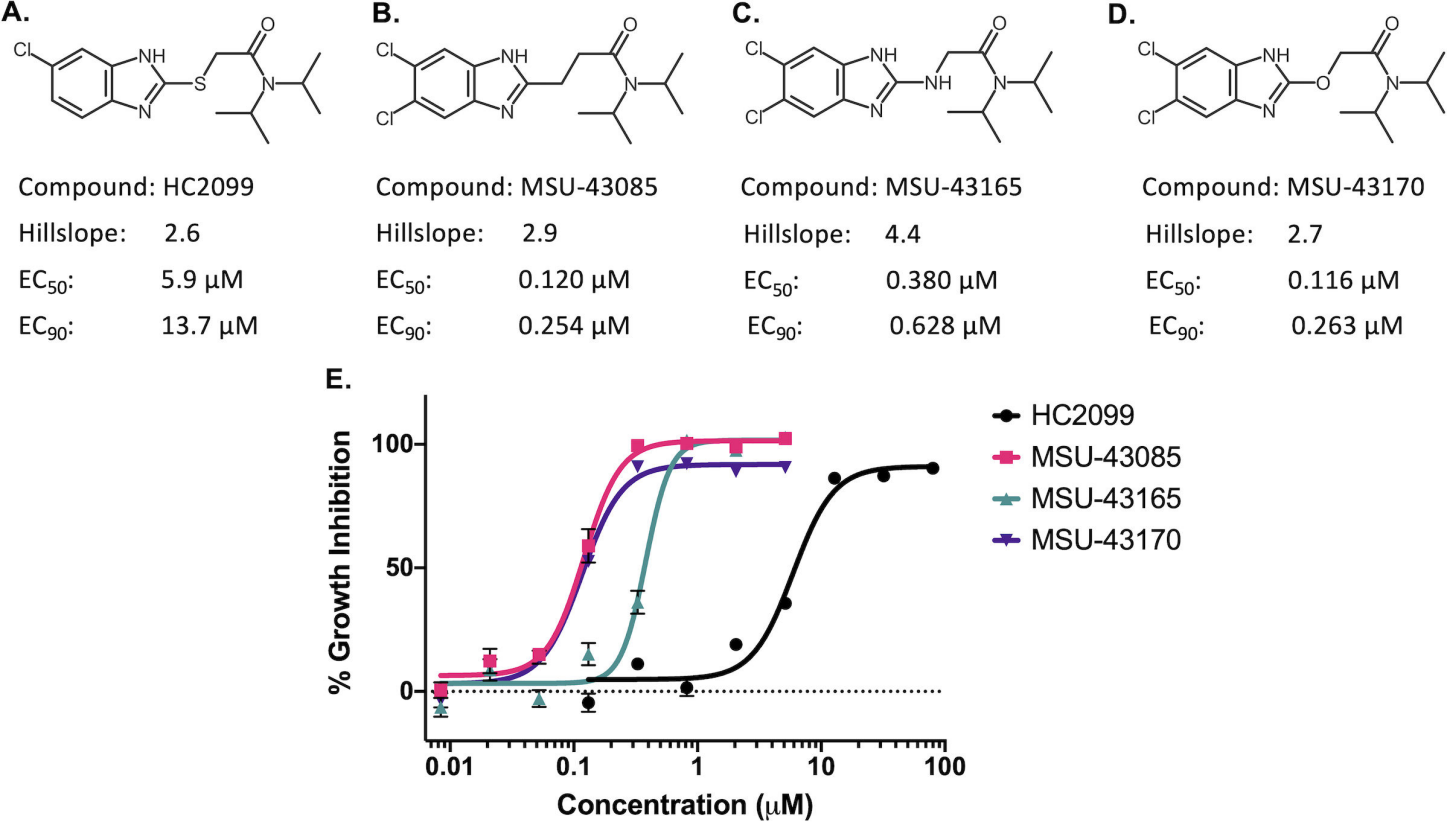
Structure-activity relationship studies enhance potency of several HC2099 analogs against Mtb. (**A–D**) Structures and activity of HC2099 (**A**) analogs MSU-43085 (**B**), MSU-43165 (**C**), and MSU-43170 (**D**). Corresponding inhibitor descriptors for dose-response curves (**E**) are included as the hillslope, EC_50_, and EC_90_. The dose-response curves for each inhibitor (**E**) are compared to parent compound HC2099 (black). Dose-response curves were run in triplicate; error bars indicate the S.D. of the mean.

In this report, we describe key derivatives from an early SAR campaign, focusing on the aromatic ring substitutions R^1^ and R^2^ (Table 1), and substitutions at “X,” to address metabolism concerns, replacing the thioether with -CH_2_- -NH-, and -O-. We have now identified multiple HC2099 analogs (Table 1) that are highly active against Mtb both *in vitro* and in macrophages. In the process, we sought to functionally characterize Mtb and MAB activities. Of these analogs, three subseries were identified, demonstrated by MSU-43085, MSU-43165, and MSU-43170 (Fig. 1). Methylation and chlorination at both R^1^ and R^2^ provided enhanced activity. This is consistent with previously described 1H-benzimidazole MmpL3 inhibitors (30). We also found that replacement of X = sulfur with oxygen, nitrogen, or carbon provided another enhancement of activity (see MSU-43065, MSU-43085, MSU-43165, and MSU-43170). The combination of modulating R^1^, R^2_,_^ and X led to ~10× −50× enhancement of activity against Mtb. The two most potent analogs (MSU-43170 and MSU-43085, EC_50_s = 120 nM) were ~10-fold higher in potency than the clinically studied MmpL3 inhibitor SQ109 but ~3- to 5-fold lower than NITD-304 and NITD-349. We prioritized these analogs for follow-up activities described below.

### HC2099 analogs kill intracellular Mtb with a high selective index

Mtb is a facultative intracellular bacterium that actively replicates in macrophages (31). Genetic knockdown studies show that MmpL3 is essential for Mtb growth inside of macrophages (32). Consistent with this phenotype, we previously reported that the parent compounds HC2099 and HC2183 analogs had potent intra-macrophage activity against Mtb (18). Using a firefly luciferase (FFLuc) reporter strain of Mtb CDC1551 (31), we screened selected active analogs for intra-macrophage activity against Mtb in infected primary bone marrow-derived macrophages (BMMΦ) via dose response (Table S1). The analogs were potent growth inhibitors of intracellular Mtb with MSU-43085, MSU-43165, and MSU-43170 exhibiting intra-macrophage EC_50_s of 134 nM, 134 nM, and 35 nM, respectively (Table S1). MSU-43170 demonstrated 3.3-fold enhanced activity compared to *in vitro* activity. Similarly, HC2099 demonstrated >10-fold enhanced activity in the same model (18). This intra-macrophage-enhanced activity was not observed for C-substituted analogs (Table S1). This indicates that both O- and S-based analogs potentially have enhanced penetration into the macrophages leading to increased intracellular Mtb killing.

To ensure that the active analogs were specifically killing Mtb and not the macrophages, we tested active analogs for cytotoxicity. The analogs demonstrated little cytotoxicity (CC_50_ >80 µM), with a selective index (CC_50_/EC_50_) range of 34.3 to >667 (Table 1). We also tested MSU-43085, MSU-42766, and MSU-43065 for cytotoxicity against THP-1, HepG2, and HeLa cell lines and found low cytotoxicity (Table S2). These results indicate that these compounds are active against intracellular Mtb without mammalian cell cytotoxicity.

### HC2099 analogs are active against NTMs

NTMs are pathogenic mycobacteria that are challenging to treat with current antibiotics (33). We previously demonstrated that parent compound HC2099 could inhibit MAB growth *in vitro* (96.5% at 20 µM) (18). To determine if the new analogs retain or improve NTM growth inhibition, we tested select analogs against NTM species MAB and MAC. For these assays, we selected analogs MSU-42766, MSU-43065, and MSU-43085, demonstrating a range of activities against Mtb with 1.3 µM, 463 nM, and 120 nM *in vitro* EC_50_s, respectively (Table 1; Fig. 1). The activity (MICs) against both MAB and MAC *in vitro* followed similar trends as activity against Mtb. Specifically, MSU-43085 was the most potent with MICs of 2.9 and 23 µM against MAB and MAC, respectively (Table 2). Comparatively, MSU-43065 demonstrated lower activities of 6.6 and 53 µM, and MSU-42766 demonstrated the lowest activity at 12 µM against MAB and no detectable activity against MAC (Table 2). While the compounds were not as potent against MAB and MAC as they are against Mtb, the activity of MSU-43085 was still higher than the standard of care drug amikacin (6.8 µM) for MAB (Table 2). Similarly, MSU-43065 had activity comparable to amikacin against MAB, while MSU-42766 had lower activity than amikacin (Table 2).

**TABLE 2:** *In vitro* and intramacrophage activity of HC2099 analogs against NTMs^*a*^

Compound	*In vitro* MIC (μM)		*Ex vivo* ( >90% inhibition, μM)	
	*M. abscessus* ATCC 19977	*M. avium* ATC 700891 (MAC 101)	*M. abscessus* ATCC 19977	*M. avium* ATC 700891 (MAC 101)
Clarithromycin	1.4	0.08	86	171
Amikacin	6.8	N.T.	N.T.	N.T.
Rifampicin	N.T.	0.07	N.T.	N.T.
MSU-42766	12	>200	>200	>200
MSU-43065	6.6	53	>200	>200
MSU-43085	2.9	23	186	47

^*a*^ N.T., not tested.

We next tested the activity of these compounds against intracellular MAB and MAC in infected macrophages. While MSU-42766 and MSU-43065 demonstrated low activity in the intra-macrophage model with MICs ≥200 µM, MSU-43085 demonstrated more potent activity against MAC (47 µM) than clarithromycin (171 µM) (Table 2).

### *mmpL3* mutants are resistant to active HC2099 analogs

We have identified analogs with enhanced activity against Mtb. However, such modifications can lead to a phenomenon, known as target drift, where new analogs target an alternative pathway from the parent compound, SQ109 serves as an example (34). Modifying the structure of EMB to SQ109, the target changed from the arabinosyl transferase EmbABC to MmpL3 (16). While target drift is not necessarily detrimental to the therapeutic potential of a new analog, such alterations in the MOA can confound SAR studies.

We previously demonstrated that a mixed mutant population of 24 unique Mtb *mmpL3* mutants, isolated against five MmpL3 inhibitors, could be used to accurately identify MmpL3 inhibitors from a small library of 163 Mtb growth inhibitors (18). This mutant pool was demonstrated to be highly resistant to MmpL3 inhibitors while demonstrating no cross-resistance to non-MmpL3 inhibitors (18). We monitored for target drift by screening active analogs via dose response against the mixed *mmpL3* mutant pool (see Table S3 for list of mutants used) and compared the area under the curve to WT Mtb Erdman. The results of this screen demonstrated that the mixed *mmpL3* mutant pool conferred 1.5- to 3.5-fold resistance (lower AUC) to all active analogs when compared to WT (Table S4; Fig. S1). MSU-43085, MSU-43165, and MSU-43170 demonstrated 1.4×, 2.0×, and 1.5× fold less activity in the *mmpL3* mixed mutant background, respectively, compared to WT (Table S4; Fig. S1). This suggests that active analogs continued to target MmpL3 and enhanced activity gained during SAR is not due to target drift.

### HC2099 analogs are active against drug-resistant Mtb strains

Mtb drug resistance contributes to treatment failure in many individuals (35). Therefore, new drugs that are active against these drug-resistant strains are needed. To determine the therapeutic potential of the compounds, we tested prioritized analogs against clinically relevant mono-drug-resistant Mtb strains including INH (*ΔkatG*), RIF (*rpoB* ^S450L^), and moxifloxacin (MOX, *gyrA* ^D94K^)-resistant strains. As expected, the INH- and MOX- resistant strains conferred resistance to INH and MOX, respectively (Table 3), and the drug-resistant strains did not confer resistance to the tested analogs MSU-42766, MSU-43065, or MSU-43085 (Table 3), consistent with other MmpL3 inhibitors (3, 6, 12, 13, 17, 36, 37). Interestingly, the activity of MSU-42766 was higher against the *rpoB* ^S450L^ strain (0.25 µg/mL) compared to WT (0.5 µg/mL) (Table 3), and the activity of MSU-43065 was higher against the *ΔkatG* and *gyrA* ^D94K^ strains (≤ 0.125 µg/mL) compared to WT (0.25 µg/mL) (Table 3). Whether background mutations confer higher susceptibility to our compounds, or Mtb strains with these specific resistance mutations are more sensitive to MmpL3 inhibitors is unknown.

**TABLE 3:** Activity of HC2099 analogs against drug-resistant *M. tuberculosis^a^*

Compound	MIC (μM)				EC_50_ (μM)	
	H37Rv	H37Rv (*rpoB* ^S450L^)	H37Rv (*ΔkatG*)	H37Rv (*gyrA* ^D94K^)	Erdman	Erdman (*mmpR5* ^E21K^)
Isoniazid	0.2	0.4	>14.5	0.2	N.T.	N.T.
Moxifloxacin	0.15	0.15	0.15	>5.0	N.T.	N.T.
Bedaquiline	N.T.	N.T.	N.T.	N.T.	0.22	0.91
Clofazimine	N.T.	N.T.	N.T.	N.T.	0.3	1.6
MSU-42766	1.5	0.8	1.5	1.5	1.3	1.8
MSU-43065	0.8	0.8	≤0.4	≤0.4	0.54	0.39
MSU-43085	≤0.3	≤0.3	≤0.3	≤0.3	N.T.	N.T.

^*a*^ N.T., not tested.

In addition to drug resistance from mutations in the target enzymes such as *gyrA* ^D94K^ for MOX or activating enzymes, such as *ΔkatG* for INH, non-specific resistance through efflux can confer resistance to some antibiotics. MmpR5 (Rv0678) is a member of the MarR family of repressors and a regulator of the *mmpS5-mmpL5* operon involved in mycobactin export and drug efflux (38). Several reports have described the *in vitro* isolation of *mmpR5* mutants resistant to bedaquiline (BDQ) and clofazimine (CFZ) (39, 40), mediated through increased MmpL5 efflux pump expression (40) and *mmpR5* clinical mutants including from BDQ and CFZ naïve patients in both drug-sensitive and MDR-TB strain backgrounds (40 – 42). In addition, Li and colleagues demonstrated that *mmpR5* mutants are resistant to the SIMBL class of MmpL3 inhibitors (43), suggesting that some MmpL3 inhibitors may be susceptible to efflux by MmpL5. To determine if *mmpR5* mutations confer resistance to our inhibitors, we tested analogs against an *mmpR5* mutant (*mmpR5* ^E21K^) isolated against an experimental compound HC2194. The *mmpR5* mutant demonstrated resistance to both BDQ and CFZ similar to previous reports (Table 3) (39 – 42, 44). However, the *mmpR5* mutant did not confer resistance to test HC2099 analogs, including MSU-43065, indicating that this series of MmpL3 inhibitors is not susceptible to MmpL5-mediated efflux (Table 3). These results show this promising series to be active against clinically relevant mono-drug-resistant and *mmpR5* efflux strains of Mtb, supporting future clinical utility.

### HC2099 analogs have a moderate frequency of resistance

The evolution of resistance to new Mtb drugs occurs at different frequencies depending on the specific drug target. For example, the prodrug INH has an *in vitro* frequency of resistance (FoR) of 10^−6^, (45) while RIF has a lower *in vitro* FoR of 10^−8^ (8) (Table 4) depending on the concentration tested. The results of cross-resistance studies against a mixed pool of *mmpL3* mutants suggested that the compounds likely target MmpL3 (Table S4) and should have a frequency of resistance (FoR) similar to other MmpL3 inhibitors (6, 9). Resistant mutants were isolated to analogs MSU-42766, MSU-43065, and MSU-43085, used in the mono-drug resistance studies (Table 3). Mutants were also isolated against controls BDQ, EMB, INH, ofloxacin (OFX), pretomanid (PRT), and RIF using increasing concentrations that were 2×, 8×, and 16× the MIC of each compound (Table 4). For all compounds tested, the FoR was relative to the concentration tested, where increasing concentrations of inhibitor led to a decrease in the FoR (Table 4). The FoR for each MmpL3 inhibitor was similar to those previously reported at higher concentrations (8, 45 – 48) (Table 4). The HC2099 analogs had similar FoR ranging from 1.36 × 10^−6^ at 2× the MIC to 4 × 10^−8^ at 8× the MIC (Table 4). No mutants were detected at the highest concentrations (16× MIC), whereas other drugs tested had a FoR ranging from 1.4 × 10^−7^ to 3.4 × 10^−8^ (Table 4). When compared to the FoR for the other drugs tested at each concentration, the FoR for the HC2099 analogs occurs at a moderate rate.

**TABLE 4:** *M. tuberculosis* frequency of resistance to HC2099 analogs^*a*^

Inhibitor	MIC (μg/mL)	Frequency of resistance		
		2 × MIC	8 × MIC	16 × MIC
Bedaquiline	2	1.43 × 10^−7^	3.57 × 10^−8^	3.57 × 10^−8^
Ethambutol	1	3.61 × 10^−6^	8.57 × 10^−7^	1.79 × 10^−8^
Isoniazid	0.125	5.0 × 10^−6^	3.93 × 10^−6^	2.71 × 10^−6^
Ofloxacin	1	5.36 × 10^−7^	7.14 × 10^−8^	3.57 × 10^−8^
Pretomanid	0.5	1.79 × 10^−6^	8.57 × 10^−7^	1.43 × 10^−7^
Rifampicin	0.5	5.36 × 10^−7^	7.14 × 10^−8^	3.57 × 10^−8^
MSU-42766	0.5	1.36 × 10^−6^	8.0 × 10^−8^	0
MSU-43065	0.25	4.0 × 10^−7^	8.0 × 10^−8^	0
MSU-43085	0.125	1.8 × 10^−6^	4.0 × 10^−8^	0

^*a*^ MIC, minimum inhibitory concentration.

### HC2099 analogs are soluble, tolerable, and orally bioavailable in mice

Based on the large selective index, and the potent whole-cell activity of the compounds both *in vitro* and intra-macrophage, we sought to determine the physical and metabolic properties of selected analogs including MSU-43085, MSU-43165, and MSU-43170 (Table 5; Table S5). To gain insights into the *in vivo* use of these series, we first tested for kinetic solubility in phosphate buffer saline (PBS) at pH 7.4 (blood relevant) and 2.0 (stomach relevant). The results of these studies note that MSU-43085, MSU-43165, and MSU-43170 have moderate solubilities at pH 7.4 (>50 to >300 µM) and increased solubility at pH 2.0 (all >300 µM). We next tested the compounds for metabolic stability in the presence of mouse microsomes. We exposed several analogs, including MSU-43085, MSU-43165, and MSU-43170, to mouse liver microsomes (S-9) for 30 min and quantified the percent remaining by mass spectrometry. Of the compounds tested, each had moderate (<85%) to high (>85%) microsome stability (Table S5). MSU-43085, MSU-43165, and MSU-43170 had high microsome stability (>95%) (Table 5).

**TABLE 5:** Snapshot PK properties of prioritized HC2099 analogs

Compound	Kinetic solubility (μM)		Microsome stability (%)	Blood plasma concentration (1 h, fold EC_50_)	Lung concentration (4 h, fold EC_50_)
	pH 7.4	pH 2.0			
MSU-43085	>75	>300	102	10–50×	8×
MSU-43165	>150	>300	106	1.3×	Present
MSU-43170	>50	>300	95	4×^*a*^	Present

^*a*^ 1-h timepoint unavailable so 2 h time point data is reported. Present, compounds detected but concentrations incalculable.

Based on these results, we performed a “snap-shot” PK study to determine if the compounds were orally bioavailable in mice. We orally dosed female C57Bl/6 mice with 200 mg/kg of MSU-43085, MSU-43165, or MSU-43170 (formulated in corn oil) and collected plasma at 1-, 2-, and 3-h post dosing and lung tissues at 4-h post dosing. Compound concentrations were quantified by UPLC/MS/MS. All three compounds were detected in both the blood plasma and lung tissue samples at 1-, 2-, and 4-h post treatment (Table 5). MSU-43085 was maximally detected at 1-h post treatment at concentrations 10–50× and 8× the EC_50_ in the blood plasma and lung tissue, respectively (Table 5). MSU-43165 was detected at a lower concentration than MSU-43085 (1.3× the EC_50_) (Table 5). For MSU-43170, usable sample volumes were not obtained for 1-h post treatment, so instead we report for 2 h at 4× the EC_50_. MSU-43165 and MSU-43170 were both detected in the lung; however, the exact concentrations could not be determined. The results of these assays suggested that the analogs could be used to orally treat Mtb in an infected mouse.

### MSU-43085 is active *in vivo* in an acute murine infection model

**Fig 2:**
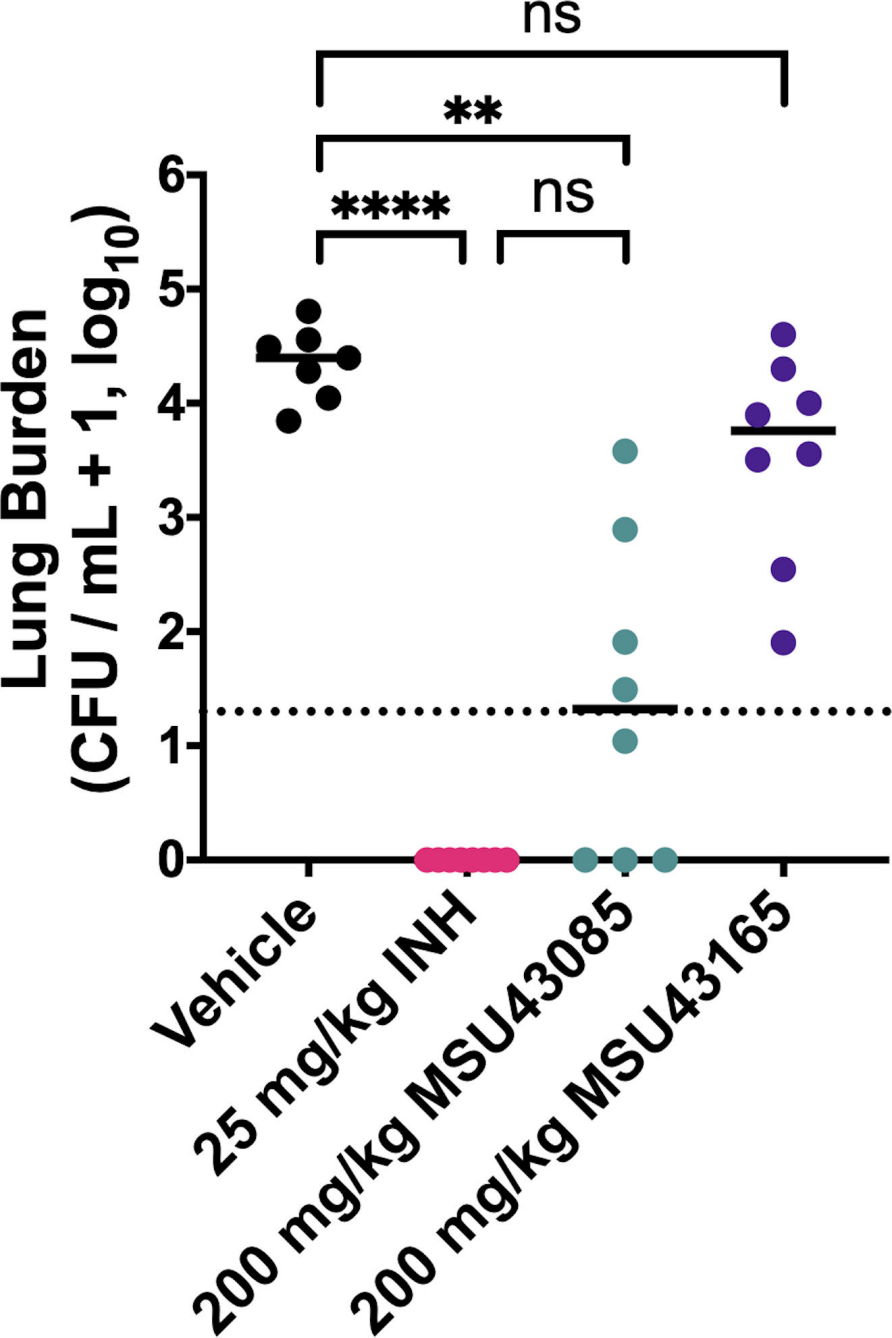
*In vivo* activity of prioritized HC2099 analogs against Mtb. (**A**) Bacterial lung burden (CFU/mL + 1) of C57Bl/6 mice following an acute (2 week) infection with Mtb Erdman and treated with vehicle (corn oil), INH (25 mg/kg), MSU-43085 (200 mg/kg), or MSU-43165 (200 mg/kg). Dotted line indicates the limit of detection (20 CFUs). CFU/mL data were +1 transformed to visualize 0 CFU data on a log scale. Solid black lines indicate the median bacterial infection in each group. Groups were compared using a two-way ANOVA. **P < 0.005, ****P < 0.0001.

Based on the potent activity against Mtb, and plasma exposure, we tested MSU-43085 and MSU-43165 in an early acute infection. For this study, we infected C57Bl/6 mice via low-dose aerosol with 200 CFUs of Mtb Erdman. In four treatment arms, the mice were treated by oral gavage, daily for 2 weeks with vehicle control (corn oil), 25 mg/kg INH, 200 mg/kg MSU-43085, or 200 mg/kg MSU-43165. The results of this acute infection model demonstrated that the lungs of vehicle control mice became well infected over 2 weeks (median = 2.2 × 10^4^ CFUs/mL) (Fig. 2). By contrast, no colonies were isolated from lung homogenates of INH-treated mice (Fig. 2). In comparison to vehicle control mice, mice treated with MSU-43085 had significantly fewer colonies (*P* = 0.0093), with a median lung bacterial burden equal to the limit of detection (20 CFUs) (Fig. 2). While colonies could still be isolated from MSU-43085 treated mice, this was not significantly different from INH-treated mice (*P* > 0.99, Fig. 2). In contrast, mice treated with MSU-43165 became well infected (median = 5.7 × 10^3^ CFUs/mL of lung), and the treatment cohort had lower overall bacterial lung burden than vehicle control mice (absolute difference of the median = 1.6 × 10^4^ CFUs/mL); this was not significant (*P* > 0.99).

Following infection in the lung, Mtb can disseminate to other tissues including the spleen. While colonies could be detected in the spleens of vehicle control mice, this was only detected in half of the infected mice (Fig. S2a). By comparison to vehicle control mice, no colonies were isolated from mice treated with either INH- or MSU-43085 (Fig. S2a). Consistent with the results for lung homogenates, MSU-43165 did not appear to prevent Mtb dissemination as colonies could also be detected in the spleens of treated mice (Fig. S2a).

Based on the results of the acute infection model, we tested the *in vivo* activity of MSU-43085 in a chronic infection model. We infected C57Bl/6 mice via low-dose (200 CFUs) aerosol and allowed the infection to establish over 4 weeks (4.7 × 10^4^ CFUs/mL) before splitting the mice into seven treatment arms that included mice treated with MSU-43085 at 200, 100, 50, and 10 mg/kg of body weight and control arms of vehicle (corn oil) or RIF (10 mg/kg). RIF and MmpL3 inhibitors, including parent compounds HC2099 and HC2183, act synergistically with RIF *in vitro (* 18, 49, 50). Therefore, we included a combination arm of mice co-treated with 10 mg/kg of RIF and 100 mg/kg MSU-43085. Mice treated with corn oil demonstrated a small decrease in CFUs over the four weeks of treatment but was not significantly different from week 4 untreated mice (Fig. S2b). In all cases of mice treated with MSU-43085, no decrease in CFU compared to week 4 untreated mice was observed (Fig. S2c). In addition, there was no significant decrease of CFUs in the co-treatment arm compared to mice treated with RIF only (Fig. S2c). While significant differences were observed between week 2 and 4 post treatment in the 200 mg/kg MSU-43085 treatment group, similar differences were observed for the vehicle control group. Therefore, conclusive interpretations could not be made from these data sets. We hypothesize that the differences in response for the 200 mg/kg between the acute infection and the chronic infection study were due to drug clearance due to unoptimized formulation. To test this hypothesis, we conducted a full PK study of MSU-43085. Although demonstrating significant *in vitro* stability against mouse microsomes and proteolytic stability, mouse IV pharmacokinetic (PK) studies (2 mg/kg) revealed a short *T*_1/2_ (~20 min, Fig. S3). To overcome this limitation, for purposes of supporting oral-dosed efficacy models, we dosed at 100 mg/kg in an oral PK study. At this concentration, we found that MSU-43085 saturated clearance, providing an observed *T*_1/2_ of 1.5 h and exposure over the EC_50_ of Mtb (120 nM) of ~11 h (Fig. S3). To further the SAR studies, metabolite ID of PK samples showed that the isopropyl methines are subject to oxidative metabolism (hydroxylation, Fig. S4), providing guidance to drive additional SAR studies.

## DISCUSSION

Early SAR studies have identified HC2099 analogs as inhibitors of MmpL3 with activity against Mtb (EC_50_) as low as 120 nM, the gain of activity resulting from substituting the S for heteroatoms C, N, or O and further substitution on the benzimidazole phenyl ring. Active analogs, including MSU-43085, MSU-43165, and MSU-43170, demonstrated potent activity against Mtb in an intra-macrophage infection model with a high selective index. In addition to Mtb, MSU-43085 demonstrates promising activities against both MAB and MAC. Selected analogs, including MSU-43085, demonstrated equal activity against both mono-drug resistant and efflux strains of Mtb. Analogs, including MSU-43085, MSU-43165, and MSU-43170, demonstrated promising PK properties and bioavailability when dosed at a high concentration. The results of an acute infection study demonstrate that MSU-43085 was active against Mtb *in vivo*. However, the results of a chronic infection model indicate that further optimization for this series is required. The compounds also demonstrated a moderate FoR. The results of these studies demonstrate that MSU-43085 is a potent inhibitor of both Mtb and MAB with promising therapeutic potential requiring additional optimization.

These early preclinical studies support the continued development of the HC2099 series for the treatment of Mtb and NTM infection. While additional *in vitro* PK studies are required to further optimize this series, one property of note is that higher solubility is observed than the leading NITD series (12). Furthermore, similar series of compounds, including the NITD, EJMCh, and CRS series, were demonstrated to be potent inhibitors of Mtb and NTMs (12, 27, 29, 51). The data reported here demonstrate that HC2099 analogs have a similar spectrum of activity against other *Mycobacterium* spp. In line with this, De Groote and colleagues recently demonstrated that a benzothiazole-based MmpL3 inhibitor (CRS400359) had higher activity against MAB compared to Mtb (51). Further studies will likely be required to design optimized inhibitors of MAB.

The *in vivo* mouse infection studies described here show that MSU-43085 can inhibit Mtb in the mouse lung during an acute infection but activity was lost in an established chronic infection. Contributing factors for this failure likely include formulation, metabolic stability, or partitioning into tissues. Recently, Lun and colleagues demonstrated that by modifying the formulation for the indole amide series of MmpL3 inhibitors, they could increase the bioavailability and increase the anti-Mtb activity of these compounds *in vivo* (52). In this study, we performed a snap-shot PK study to establish that the compounds are bioavailable; the results demonstrated that MSU-43085 was present both in blood plasma and lung tissue and was backed by the *in vivo* activity for MSU-43085 against Mtb in an acute infection. However, the full PK parameters for this study showed a short-half life for the compound due to metabolism of the isopropyl methines. Therefore, additional studies are required to understand the pharmacokinetics of this series to guide further optimizations, formulation, and dosing schedules. During the *in vivo* studies, we did not observe any behavioral changes or weight loss in mice treated with MSU-43085, even when taken for 4 weeks at a high dose (200 mg/kg). This demonstrates the compound is tolerable. However, additional studies into the potential side effects and toxicity of this series of inhibitors still need to be conducted, particularly for a compound with a longer half-life. Notably, the high-potency and short-half life could make MSU-43085 an ideal agent for inhaled formulations to treat Mtb or NTM infections.

## MATERIALS AND METHODS

### Culture conditions and strains

Mtb strains were cultured in 10 mL 7H9 OADC and 0.05% Tween-80 in standing T25 flasks. Cultures were incubated at 37°C in 5.0% CO_2_. Cultures were aliquoted into 2 mL screw cap tubes and mixed 1:1 with 40% glycerol. Mtb-glycerol tubes were stored in a −80°C freezer. When needed, stocks were thawed at room temperature and inoculated into 30 or 100 mL cultured of 7H9 OADC (10%, vol/vol) with 0.05% Tween-80 in standing T75 or T150 flasks, respectively, and grown to exponential phase (OD_600_ = 0.5–1.0).

For the mixed mutant pool of *mmpL3* mutants, 24 unique Mtb *mmpL3* mutants were pooled in an equal density mixture based on OD_600_ as previously described (18). The pooled mutant culture was then aliquoted into 2 mL cryo-tubes and mixed 1:1 with 40% glycerol (20% glycerol final) and stored at −80°C. For a full list of *mmpL3* mutants used, see Table S3. Cryo-tubes were thawed as described above and inoculated into 30 mL or 100 mL of 7H9 medium in T75 or T150 standing flasks and incubated at 37°C with 5.0% CO_2_.

*M. abscessus* (ATCC19977) and *M. avium* (ATCC 700891–MAC 101) were cultured on solid medium plates, and CFUs were taken to inoculate 7H9 ADC with 0.05% Tween-80 and supplemented with glycerol (2 mL/L) liquid cultures. Liquid cultures were incubated at 35–37°C

### Compound synthesis and authentication

Details on the methods and authentication of synthesized compounds are described in the supplemental materials.

### *In vitro* dose responses

Half maximal effective concentrations (EC_50_) dose-response curves were conducted as previously described (18, 20). Briefly, Mtb strains were grown to mid-log phase and seeded in 96 or 384 well plates at a starting OD_600_ of 0.1 or 0.05 for 384 well. Wells of bacteria were then treated with each compound in a range of 80–0.13 µM or 5.12 µM–8.3 nM (2.5-fold dilutions, for eight dose points) as well as DMSO (negative) and 0.3 µM RIF (positive) controls. Plates were then placed into re-sealable bags with a wet paper towel and incubated at 37°C with 5.0% CO_2_ for 6 days. After 6 days, plates were removed, and bacterial wells were resuspended by pipetting. Growth (OD_600_) was analyzed using a PerkinElmer Enspire plate reader, and percent growth inhibition (%GI) was calculated relative to DMSO and RIF controls. Dose responses were log transformed and used to calculate EC_50_s, Hillslopes, EC_90_s, 95% confidence intervals (CI), and area under the curves (AUCs) using Graphpad Prism 9 software.

The MIC of the compounds and drugs tested was determined using the microbroth dilution method. Briefly, bacterial strains were grown to logarithmic phase and seeded into 96 wells plates at 5 × 10^5^ CFU/mL. Cells were then treated with each compound (32–0.12 µg/mL) or control drugs RIF (1–0.004 µg/mL), INH and MOX (2–0.008 µg/mL), and Pretomanid (8–0.03 µg/mL). Bacterial plates were then sealed and incubated at 37°C for 7–8 days. Following OD_600_ measuring, 10 µL of Alamar blue dye was added and scanned on a flatbed color. The MIC for optical density was defined as the first concentration to decrease the OD_600_, while the MIC for the calorimetric assay was calculated as the first concentration for the observed color change form pink (active growth) to blue (no growth).

### *mmpL3* cross resistance profiling

Dose-response curves were generated as described above. Area under the curve (AUC) was calculated in Prism 9 software and compared between the mixed mutant pool and Mtb Erdman strains. Relative AUCs were calculated by dividing the AUC of the mixed *mmpL3* pool by the WT Erdman AUC. Fold resistance was calculated as the inverse of the relative AUCs.

### Isolation and sequencing of an *mmpR5* Mtb mutant

*mmpR5* mutants were isolated, screened, and sequenced as previously described (18). Briefly, Mtb Erdman was cultured to exponential phase in 7H9 medium. 1 × 10^9^ CFUs were harvested and plated on a 150 mm 7H10 OADC plate (with 10 µg/mL cycloheximide) amended with 20 µM of experimental compound HC2194. Plates were incubated at 37°C until single colonies appeared. Strains were colony purified to remove potential contaminating WT and screened for resistance by dose response. The genomes of confirmed resistant mutants were isolated and sequenced as previously described (18). Sequenced genomes of resistant mutants were analyzed using the GATK workflow to identify single-nucleotide variations (53).

### Sensitivity testing against drug resistant and an *mmpR5* efflux mutant

The MICs and EC_50_ were determined as described above. Sensitivity testing was compared between mono-drug-resistant strains *rpoB* ^S450L^ (RIF^R^), *ΔkatG* (INH^R^), and *gyrA* ^D94K^ (MOX^R^) strains as well as against an *mmpR5* ^E21K^ efflux pump mutant. EC_50_s were considered significantly different if the EC_50_ 95% CI did not overlap.

### Intracellular EC_50_ dose-response curves

Intracellular dose-response curves were carried out as previously described (54). Briefly, a firefly luciferase (FFluc) reporter strain of Mtb CDC1551 (55) was grown to mid-log phase. Cultured resting primary bone marrow-derived macrophages were isolated from C57Bl/6 mice and seeded into 96 well plates as described (54). Macrophages were then infected with the reporter strain at a MOI of 1 (54). Wells were then treated in duplicate with selected compounds in a range of either 80 µM–8.3 nM or 5.12 µM–8.3 nM (8 or 12 dose points, 2.5-fold dilutions). DMSO and 0.3 µM RIF were included as controls. 96 wells plates were then incubated in vented re-sealable bags with a wet paper towel at 37°C with 5.0% CO_2_ for 6 days. After 6 days, the Bright-Glo luciferase agent (Promega) was added to the 96 wells plates in a 1:1 ratio for each well. Plates were then read using a PerkinElmer Enspire plate reader. Percent intracellular growth inhibition was calculated relative to DMSO and RIF controls. EC_50_s, Hillslopes, and EC_90_s were calculated as described above using Graphpad Prism 8 Software.

### Cytotoxicity

Macrophage cytotoxicity for tested analogs was calculated for BMMΦ using the Cell Titer Glow (Promega) assay as previously described (54). Briefly, BMMΦ were harvested from mice and seeded in 96 well plates at a starting inoculum of 1 × 10^7^ cells/mL. Wells were treated with select compounds at a range of 80 µM to 8.3 nM in duplicate (12 dose points, with 2.5-fold dilutions). DMSO and 4.0% Triton X-100. 96 well plates were incubated at 37°C with 5.0% CO_2_ in vented re-sealable bags with a wet paper towel. After 6 days, wells were treated 1:1 with CellTiter-Glo reagent and plates were read using a PerkinElmer Enspire plate reader. Percent cytotoxicity was calculated relative to DMSO (negative) and Triton (positive) controls. Transformed % cytotoxicity was used to calculate the CC_50_ using Graphpad Prism 8 Software.

For additional cell lines including HepG2, HeLa, and THP-1, the following protocol was used. HepG2 (ATCC HB-8065) and HeLa (ATCC CCL-2) were cultured in cEMEM supplemented with 10% (vol/vol)/FBS and 1%(vol/vol) PenStrep. THP-1 cells were cultured in cRPMI-1640 medium (ATCC 30-2001) supplanted with 10% (vol/vol) FBS and 0.05 mM 2-mercaptoethanol (Sigma M3148). THP-1 cells were seeded in 96 well plates at 1 × 10^5^ cells/mL with complete medium. HepG2 and HeLa cells were seeded in 96 well plates with cEMEM at 1 × 10^6^ cells/mL. Cells were treated with compounds by dose response in a range of 128–0.48 µg/mL in triplicate or mitomycin C at a range of 128–0.0625 µM. Cells were treated for 24 h at 37°C with 5% CO_2_. Cells were then treated with either MTT or resazurin for 4 h. Absorbance was then measured at 570 nm for MTT plates. Percent cytotoxicity was calculated relative to positive mitomycin C and untreated controls. Percent cytotoxicity was used to calculate the IC_50_ using Graphpad prism software using non-linear regression analysis.

### *In vitro* and intra-macrophage activity against NTMs

The MICs for *M. avium* (MAC 101) and *M. abscessus* (ATCC 19977) were calculated as above with minor alterations. Bacteria were suspended in cation-adjusted Mueller Hinton broth and seeded in 96 well plates at 5 × 10^5^ CFUs/mL. Cells were then treated with compounds as well as clarithromycin and amikacin at concentrations ranging from 32 to 0.12 µg/mL. Plates were then sealed and incubated for 7 days at 37°C. Optical density and calorimetric readings were then taken as described above. MICs were defined as above.

THP-1 cells were infected with either *M. avium* or *M. abscessus* at an MOI of 10 for 4 h prior to treatment. Following infection, cells were washed twice with PBS to remove non-phagocytosed bacteria. Fresh medium was then added, and cells were treated with concentrations ranging from 64 to 0.24 µg/mL and incubated for 24 h at 37°C with 5% CO_2_. Following 24 h of treatment, cells were treated with resazurin (0.04 mg/mL) and incubated for an additional 3–5 days. Absorbance was measured for each well at 570 and 600 nm. The percent growth reduction was calculated relative to treated and untreated controls.

### Kinetic solubility and microsomal stability assays

The kinetic solubility assay was conducted as previously described (18). Briefly, using methods described by Bevan and Lloyd (56), the assay was performed with 7-point (twofold) dilutions of the compounds, from 200 to 3.125 μM along with mebendazole, bexarotene, and aspirin as controls. The drug dilutions were added to PBS, pH 7.4, with a DMSO concentration of 1% (final), and incubated at 37°C for 2 h. The absorbance at 620 nm was measured for each drug dilution to estimate the solubility of the compound in triplicate/ dilution.

The mouse microsomal stability assay was conducted as described by Obach (57), and the results are presented as the percentages remaining after 30 min. Values greater than 100% are likely due to changes in the solubility of the compounds over the course of the assay and represent high stability in microsomes.

### Frequency of resistance

Freezer stocks of Mtb H37Rv were thawed and inoculated into 7H9 OADC and grown to stationary phase (OD_600_ of ~1.0) at 37°C rotating at 150 rpm. Samples were sub-cultured 1:100 in 100 mL of fresh 7H9 OADC and incubated at 37°C rotating at 150 rpm to an OD_600_ of ~1.0 (i.e*.,* ~1 × 10^8^ CFU/mL). 0.2 mL aliquots of sub-culture adjusted Mtb (OD_600_ of 1.00) were plated onto 7H10 ADC agar plates amended with or without (control) tested inhibitors (Table 4) at 2-, 8-, and 16 times the MIC. Plates were incubated at 37°C for 3–4 weeks to enumerate resistant mutant colonies (CFU). After incubation, colonies were inoculated into 4 mL of 7H9 containing equivalent to concentrations tested and cultured at 37°C rotating at 150 rpm for 2 to 3 weeks to confirm resistance. The FoR was calculated as the number of confirmed resistance colonies compared to the number of colonies initially plated (unamended control plate). The FoR range was from 1 to 1 × 10^−8^.

### PK studies

All experiments were performed in compliance with Michigan State University IACUC approved protocols.

#### Short PK studies

Female C57Bl/6 mice were dosed with 200 mg/kg (based on the average body mass of 27 mice) of MSU-43085, MSU-43165, or MSU-43170 formulated in corn oil by oral gavage. At 1-, 2-, and 4-h post treatment, blood samples were taken from three mice from each treatment group by cardiac puncture and placed in BD Lithium HeparinN Vacutainer tubes. Plasma was separated from whole blood by centrifuge, and compounds were methanol extracted. Lungs were harvested from mice 4- h post treatment. Lung samples were homogenized using a closed tissue grinder system (Fisherbrand—02-542-09), and compounds were methanol extracted from lung homogenates. Methanol-extracted samples were quantified by UPLC/MS/MS.

#### IV PK study MSU-43085

Similar to what was completed for short PK studies, female C57Bl/6 mice were dosed IV with 2 mg/kg (based on the average body mass of 28 mice) of MSU-43085, formulated in 0.5% each of DMSO and Tween-80 in PBS buffer. At 0.08, 0.5, 1.0, 2.0, 8.0, and 24 h (four animals per treatment group), blood samples were taken by cardiac puncture and placed in BD Lithium HeparinN Vacutainer tubes. Samples were processed for analysis as described above, quantified by UPLC/MS/MS.

#### Oral PK study (100 mg/kg) MSU-43085

As what was completed for short PK studies, female C57Bl/6 mice were dosed orally with 100 mg/kg (based on the average body mass of 28 mice) of MSU-43085, formulated in corn oil. At 0.25, .0.5, 1.0, 2.0, 8.0, 12.0, and 24 h (four animals per treatment group), blood samples were taken by cardiac puncture and placed in BD Lithium HeparinN Vacutainer tubes. Samples were processed for analysis as described above, quantified by LC/MS/MS.

### Metabolite identification studies

To assess the metabolic process for the clearance of MSU-43085, NERDD (58) was used as an E-resource to identify likely potential metabolites. Using the samples produced from PK studies, we identified each of the metabolites and their relative timing to be produced by UPLC/MS/MS using the 0.25-, 0.5-, 1.0-, and 2-h timepoints. Structures were verified by observed MS fragmentation patterns.

### Acute mouse infection model

Forty 6- to 8-week-old female C57Bl/6 mice were infected with Mtb Erdman via low-dose aerosol (200 CFUs) using a Glas-col nebulizer. One day post infection (P.I.), whole lungs from untreated mice were harvested from euthanized mice and homogenized using a Next Advance Bullet Blender in PBS with 0.1% Tween-80 using 1.6 mm diameter steel beads. Samples of lung homogenates were plated in duplicate on 7H10 agar plates supplemented with 10% v/v OADC with 100 µg/mL cycloheximide, 50 µg/mL carbenicillin, 25 µg/mL polymyxin B, 20 µg/mL trimethoprim and incubated at 37°C for 40 days. Starting one day post infection, the remaining 32 mice were separated into four treatment arms of eight mice each. Mice from each arm were treated 5 days per week (over a total of 12 days) with either vehicle control (Corn oil), 25 mg/kg INH (in H_2_O), 200 mg/kg MSU-43085, or 200 mg/kg MSU-43165 melted into corn oil. Thirteen days P.I., mice were euthanized as described above, and spleens and right lung lobes were homogenized as described above. Tissue homogenates were serial diluted in PBS (pH 7.4) +0.05% Tween-80 and plated in duplicate onto 7H10 plates. Plates were incubated for 40 days at 37°C and CFUs enumerated. Left lung lobes were fixed in 4% paraformaldehyde.

No colonies were isolated from four out of the eight spleen samples from untreated mice; therefore, no statistical tests were performed on these samples. No colonies were detected in samples from one of the untreated control mice even in undiluted samples. A Grubb’s outlier test of Log_10_ transformed CFUs of the untreated control group indicated that this mouse was an outlier (*P* < 0.01); therefore, this mouse was removed from downstream analysis. Bacterial burden was compared between treatment groups using a one-way ANOVA with Kruskal-Wallis post hoc test.

### Chronic mouse infection model

Female C57Bl/6 mice were infected by low-dose aerosol (200 CFUs) with Mtb Erdman as described above. One day P.I., eight mice were euthanized, and CFUs were enumerated from lung homogenates as described above. For the remaining 120 mice, Mtb infection was left to establish for 4 weeks before being broken into treatment arms. Following 4 weeks of infection, eight mice were euthanized, and lung and spleen samples were homogenized as described above to quantify infection prior to treatment. The remaining mice were broken into 7 treatment arms of 16 mice each and treated 5 days per week over 4 weeks. Treatment arms consisted of a vehicle control (100 µL corn oil); 10 mg/kg Rif (in 200 µL H_2_O); 10, 50, 100, or 200 mg/kg MSU-43085 (in 100 µL corn oil); or a combined treatment of 10 mg/kg RIF (in 200 µL H_2_O) and 100 mg/kg MSU-43085 (in 100 µL corn oil). At 6 and 8 weeks P.I., eight mice from each treatment group were euthanized, and spleen and right lung lobes were homogenized as described above. Lung and spleen homogenates were serial diluted in PBS (pH 7.4) with 0.05% Tween-80 and inoculated onto 7H10 antibiotic plates. Plates were incubated for up to 40 days at 37°C and CFUs enumerated.

Body weights (g) of mice were measured to the nearest 0.5 g at the start of treatment and were measured once a week throughout the experiment. The changes in percent body weight were calculated for each mouse relative to body weights measured prior to treatment. A positive gain indicates a gain in weight. The average body weights measured per group were used as the basis for dosing the mice each week.

## SUPPLEMENTAL MATERIAL

Supplemental material spectrum.03677-23-s0001.pdf Tables S1 to S5, Figures S1 to S4, and supplemental methods.

ASM does not own the copyrights to Supplemental Material that may be linked to, or accessed through, an article. The authors have granted ASM a non-exclusive, world-wide license to publish the Supplemental Material files. Please contact the corresponding author directly for reuse.
