# Supplementary material for: The Mycobacterium tuberculosis MmpL3 inhibitor MSU-43085 is active in a mouse model of infection
Source: Microbiol Spectr. 2023 Dec 11;12(1):e03677-23. doi: 10.1128/spectrum.03677-23 (PMC10783087; doi:10.1128/spectrum.03677-23)
Supplement: Supplemental material — Tables S1 to S5, Figures S1 to S4, and supplemental methods. [file spectrum.03677-23-s0001.pdf]

Supplemental Figure Legends

Figure S1. Cross resistance of a mixed Mtb *mmpL3* mutant pool against select analogs. a-c) Dose response curves for compounds MSU-43085 (a), MSU-43165 (b), and MSU-43170 (c) in either a wild type (WT) or mixed *mmpL3* mutant background. Fold differences in the effective concentration are listed as the area under the curve in Table 3. *mmpL3* mutant strains used are listed in Table S3.

Figure S2. Spleen burden of Mtb acute infection. (a) Spleen bacterial burden of C57Bl6 mice following an acute (two week) infection with Mtb Erdman and treated with vehicle (corn oil), INH (25 mg/kg), MSU-43085 (200 mg/kg) or MSU-43165 (200 mg/kg). No detectable colonies were isolated from most vehicle control mice. Solid black line indicates the median burden. Due to inconsistency of infection, statistical tests were not performed. b) Bacterial lung burden (CFU/mL) of C57Bl/6 mice in a chronic infection model. Following four weeks of infection, mice were split into seven treatment groups and treated for two- and four- weeks via p.o. c) Bacterial lung burden (CFU/mL) of mice at week 8. Significant differences were observed between treatment groups based on a two-way ANOVA.

Figure S3. PK studies of MSU-43085. A. IV PK (2 mg/kg) of MSU-43085 measuring plasma concentration. B. Oral PK measuring plasma concentrations (100 mg/kg) dosing relative to the EC50 of MSU-43085.

Figure S4. Metabolism of HC43085 in orally treated mice.

Table S1. Activity of select HC2099 analogs against intracellular *M. tuberculosis*

Compound Name	*ex vivo* EC_{50} (μM)	Hillslope	*ex vivo* EC_{90} (μM)	BMMΦ CC_{50} (μM)	S.I. (CC_{50} / EC_{50})
MSU-42766	0.41	2.5	0.97	> 80	> 195.6
MSU-43065	0.29	17.8	0.33	> 80	> 274.3
MSU-43085	0.13	1.8	0.46	> 80	> 595.7
MSU-43086	0.33	21.3	0.37	> 80	> 238.8
MSU-43165	0.13	16.5	0.15	> 80	> 596.6
MSU-43166	0.6	6.2	0.86	> 80	> 132.2
MSU-43170	0.035	1.6	0.14	> 80	> 2285

BMMΦ – Primary bone marrow derived macrophages, CC_{50} – Cytoxicity of compounds against , S.I. – Selective index

Table S2. Cytotoxicity of select HC2099 analogs against additional cell lines

Compound Name	THP-1 Cytotoxicity (IC_{50} μg/mL)	HepG2 Cytotoxicity (IC_{50} μg/mL)	HeLa Cytotoxicity (IC_{50} μg/mL)
MSU- 42766	39.6	> 128	> 128
MSU- 43065	69.51	> 128	> 128
MSU- 43085	> 128	> 128	> 128
Mitomycin C	3.094	23	10.54

Table S3. ***M. tuberculosis mmpL3*** **mutants used in pooled mutant cross resistance study**

Compound Background	Mtb Strain Background	Strain	SNP Location	Quality Score	Gene	Nucleotide Change	Amino Acid Substitution
**HC2060**	Erdman	1A	245506	5308	*mmpL3*	GTG --> ATG	V643M
	Erdman	3B	245501	4710	*mmpL3*	TTC --> TTG	F644L
	Erdman	4A	245733	3840	*mmpL3*	CTG --> CCG	L567P
	Erdman	5B	245501	4807	*mmpL3*	TTC --> TTA	F644N
	Erdman	6C	245349	4795	*mmpL3*	ATG --> ACG	M695T
**HC2091**	CDC 1551	3A	245488	3441	*mmpL3*	ATG --> CTG	M649L
	CDC 1551	5A	245424	2685	*mmpL3*	ACC --> AAC	T670L
	CDC 1551	23A	245335	2615	*mmpL3*	GCC --> ACC	A700T
**HC2149**	Erdman	1A	245487	5798	*mmpL3*	ATG --> ACG	M649T
	Erdman	3A	247313	5895	*mmpL3*	CAG --> CAT	Q40H
	Erdman	11C	246316	5127	*mmpL3*	CGG --> TGG	R373W
	Erdman	15A	246501	3836	*mmpL3*	ACC --> ATC	T311I
	Erdman	16A	246537	3854	*mmpL3*	CTG --> CAG	L299Q
**HC2169**	Erdman	1B	245662	7164	*mmpL3*	TCG --> ACG	S591T
	Erdman	14A	246579	4739	*mmpL3*	GTG --> GGG	V285G
	Erdman	13A	246675	4585	*mmpL3*	GGG --> GAG	G253E
	Erdman	7A	246678	6076	*mmpL3*	TAC --> TGC	Y252C
	Erdman	16A	246702	5280	*mmpL3*	ATC --> ACC	I244T

Table S3(cont'd)

**HC2184**	Erdman	1B	245355	5122	*mmpL3*	GAC --> G**G**C	L693P
	Erdman	6B	245661	5719	*mmpL3*	TCG --> T**A**G	S591I
	Erdman	8A	246678	5654	*mmpL3*	ATG --> A**C**G	I585S
	Erdman	12B	245338	5281	*mmpL3*	GAC --> **T**AC	L699M
	Erdman	13B	245448	5121	*mmpL3*	CGC --> C**T**C	A662E
	Erdman	20A	246714	3800	*mmpL3*	CAC--> C**G**C	V240A

Mtb – *M. tuberculosis*, SNP – single nucleotide polymorphism

Table S4. Cross resistance of active HC2099 analogs against a mixed *mmpL3* mutant pool

Compound Name	WT AUC	Mixed *mmpL3* AUC	Fold Resistance (WT / *mmpL3*)
HC2183	101.7	34.08	2.92
MSU-42766	169.9	73.44	2.35
MSU-43065	204.8	133.1	1.54
MSU-43085	231.7	165.2	1.41
MSU-43165	214.5	147.2	1.45
MSU-43170	247.5	160.3	1.54

AUC – area under the curve

Table S5. Early pharmacokinetic properties of prioritized HC2099 analogs

Compound Name	cLogP	Kinetic solubility at pH 7.4 (µM)	Kinetic solubility at pH 2.0 (µM)	Microsome Stability (%)
HC2099	3.7	178	> 300	71
HC2183	3.6	> 200	> 300	25
MSU-43085	3.5	> 75	> 300	102
MSU-43165	3.3	> 150	> 300	106
MSU-43170	3.2	> 50	> 300	95

% - % of compound remaining after 30 minutes of incubation with mouse microsomes, N.T. – not tested

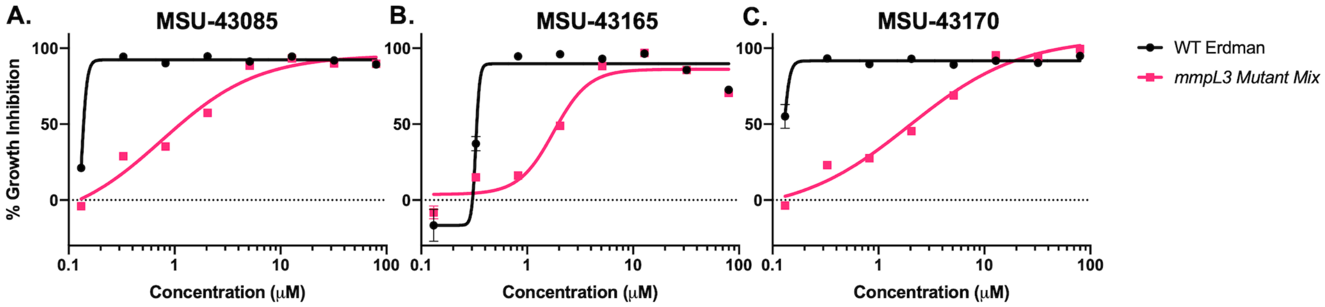

Supplemental Figure 1

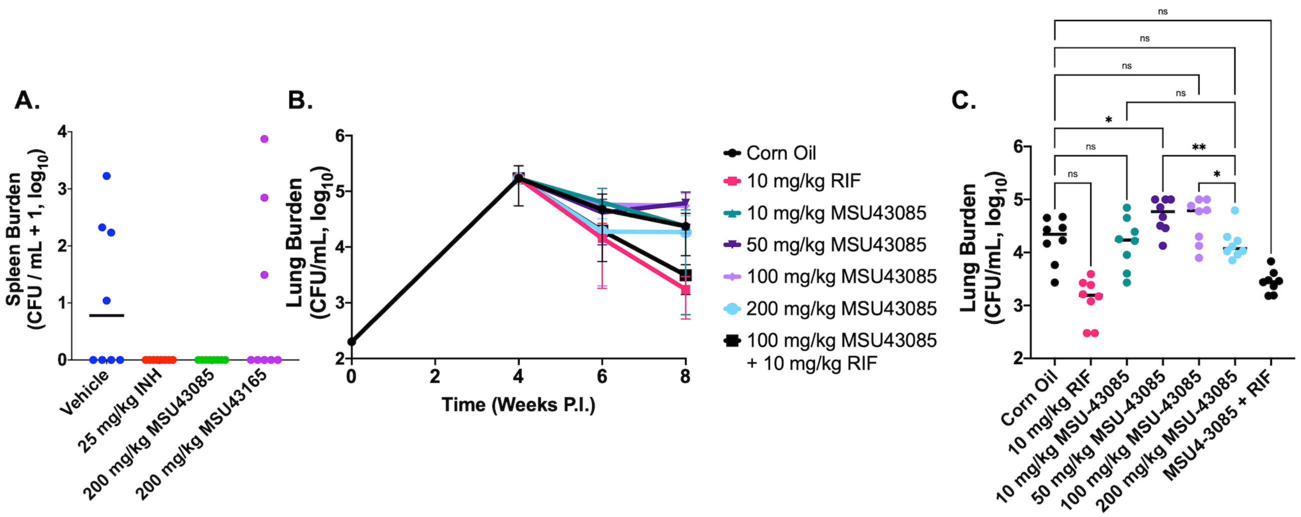

Supplemental Figure 2

A

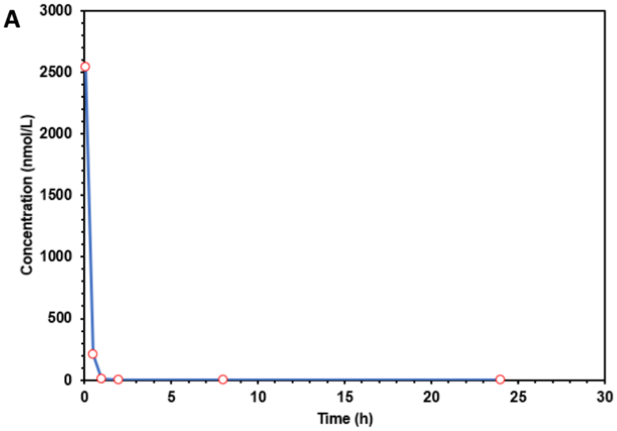

IV PK (2 mg/kg, Mice)) of MSU-43085 plasma concentrations.

Parameter	Value
$T_{1/2}$ (hr.)	0.15
T_{max} (hr.)	0.06
C_{max} (nmol/L)	1407
AUC 0-inf_obs (nmol/L*h)	527
Vz obs (L/kg)	0.74
CL (L/hr)	6.9

B

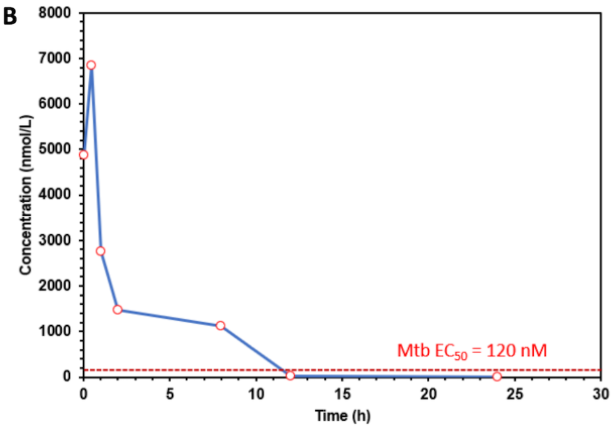

Oral PK (Mice) measuring plasma concentrations (100 mg/kg) dosing relative to the EC_{50} of MSU-43085.

Parameter	Value
$T_{1/2}$ (hr.)	4.5
T_{max} (hr.)	2
C_{max} (nmol/L)	2490
AUC 0-inf_obs (nmol/L*h)	18497

When dosed at 100 mg/kg, time above the EC_{50} ~11 hours.

Supplemental Figure 3

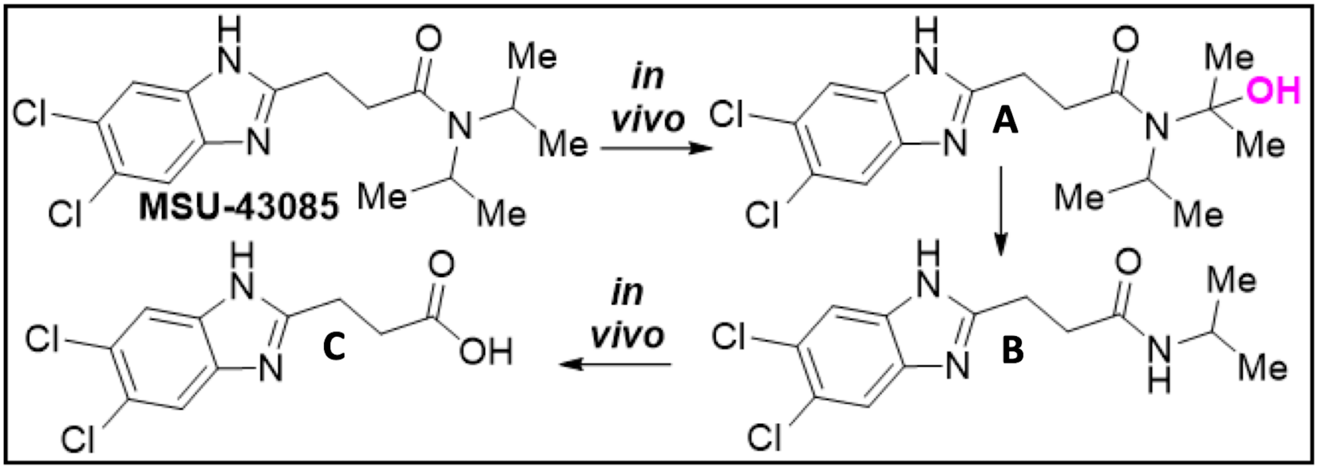

Compound	Molecular weight - Exact (g/mol)	Exact Mass [M+H] Observed (g/mol)
**MSU-43085**	341.106	342.114
**A**	357.101	358.109
**B**	299.059	300.067
**C**	257.996	259.004

Supplemental Figure 4

Supplemental Methods

HC2099 analogs synthesis

Scheme 1. Preparation of MSU-42766

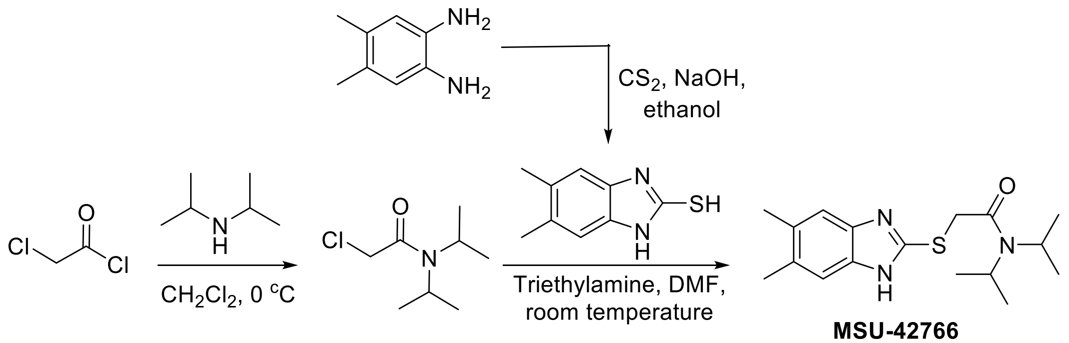

2-chloro-N,N-bis(propan-2-yl)acetamide. The title compound was prepared by the following method (Chen, T. C.; Yu, D. S.; Fu, Y. C.; Lee, C. C.; Chen, C. L.; Huang, F. C.; Hsieh, H. H.; Lin, J. J.; Huang, H. S., *Eur. J. Med. Chem*, **2013**, *69*, 278). A 100 mL 24/40 round bottom flask (argon atmosphere) was charged with dichloromethane (20.0 mL) and chloroacetyl chloride (1.12 g, 10.0 mmol) and cooled to 0 ºC. Diisopropylamine (2.52 g, 25.0 mmol), dissolved in dichloromethane (10.0 mL), was added (0 ºC) to the rapidly stirring solution, in a dropwise fashion. resulting in a precipitate. The mixture was stirred for 30 minutes at 0 ºC, then treated with 30.0 mL of a 1.00 N aqueous hydrochloric acid solution. The biphasic reaction mixture was stirred for 5 minutes at 0 ºC, then transferred to a separatory funnel and partitioned. The aqueous layer was washed with 15.0 mL dichloromethane and combined with the organic layer. The organic mixture was washed with brine, dried over sodium sulfate, filtered, and concentrated *in vacuo* to yield an oil, which was filtered through a plug of silica (100 % dichloromethane). The filtrate was concentrated *in vacuo* to yield the pure product as a clear, colorless oil (1.51 g, 85 % yield). ^{1}H NMR (500 MHz, $CDCl_3$) δ 4.01 (s, 2H), 3.95 (p, J = 6.7 Hz, 1H), 3.43 (dd, J = 13.1, 6.9 Hz, 1H), 1.39 (d, J = 6.8 Hz, 6H), 1.24 (d, J = 6.7 Hz, 6H).

5,6-dimethyl-1H-1,3-benzodiazole-2-thiol. A 100 mL 24/40 round bottom flask was charged with ethanol (20.0 mL), carbon disulfide (1.67 g, 22.0 mol) and solid sodium hydroxide (0.880 g, 22.0 mmoles). 4,5-dimethylbenzene-1,2-diamine (2.58 g, 19.0 mmol) was added, followed by 3.00 mL water. The reaction mixture (argon atmosphere) was heated to reflux for 3 hours and a precipitate formed. The reaction mixture was cooled to room temperature, filtered, and washed with ethanol. The filtrate was diluted with 20.0 mL water, heated to 70 ºC with stirring, and treated with 10.0 mL of a 1:1 acetic acid and water mixture, resulting in the formation of a precipitate. The mixture was cooled to 0 ºC for 3 hours, filtered, and dried *in vacuo* for 20 hours to yield a solid (2.50 g, 74 %, prepared according to the method described by Peddibhotla, S.; Shi, R.; Kahn,

P.; Smith, L. H.; Mangravita-Novo, A.; Vicchiarelli, M.; Su, Y.; Okolotowica, K. J.; Cashman, J. R.; Reed, J. C.; Roth, G. P.; *J. Med. Chem.*, **2010**, *53*, 4793.). ^{1}H NMR (500 MHz, $CDCl_3$) δ 7.47 (dd, *J* = 6.0, 3.2 Hz, 1H), 7.17 (dd, *J* = 6.0, 3.1 Hz, 1H), 3.61 (s, 1H), 3.33 (h, *J* = 6.5 Hz, 1H), 1.34 (d, *J* = 6.5 Hz, 6H).

2-[(5,6-dimethyl-1H-1,3-benzodiazol-2-yl)sulfanyl]-N,N-bis(propan-2-yl)acetamide (MSU – 42766).. A 50.0 mL round bottom flask was charged with 2-chloro-N,N-bis(propan-2-yl)acetamide (0.354 g, 2.00 mmol), dry dimethylformamide (2.00 mL), and 5,6-dimethyl-1H-1,3-benzodiazole-2-thiol (1.00 mmol, 0.178 g). The reaction mixture was stirred, under an argon atmosphere, and treated with triethylamine (0.252 g, 2.50 mmol). After 18 hours, the mixture was poured into water (50.0 mL), and the resulting solid filtered, washed with water and hexanes, then dried *in vacuo* to provide 2-[(5,6-dimethyl-1H-1,3-benzodiazol-2-yl)sulfanyl]-N,N-bis(propan-2-yl)acetamide (0.161 g, 50 %). ^{1}H NMR (500 MHz, DMSO-d_6) δ 12.23 (s, 1H), 7.24 (s, 1H), 7.11 (s, 1H), 4.35 (s, 2H), 4.06 (p, *J* = 6.6 Hz, 1H), 3.52 – 3.45 (m, 1H), 2.25 (d, *J* = 6.7 Hz, 6H), 2.07 (s, 2H), 1.27 (d, *J* = 6.7 Hz, 6H), 1.17 (d, *J* = 6.6 Hz, 6H). APCI HRMS [M+H] calc'd = 320.1791, found 320.1790. m.p = 172 °C.

Scheme 2. Preparation of MSU-43065

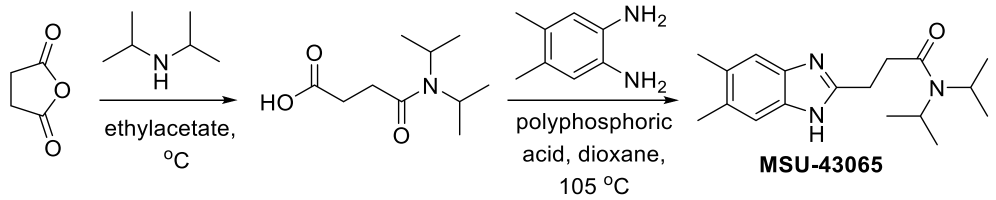

3-[bis(propan-2-yl)carbamoyl]propanoic acid. A 100 mL 24/40 round bottom flask was charged with succinic anhydride (2.00 g, 19.0 mmol) and ethyl acetate (20.0 mL) and treated dropwise with diisopropylamine (3.08 mL, 22.0 mmol). The mixture was heated to reflux for 23 hours, then concentrated *in vacuo* to yield the product as an oil (3.8 g, 100 %). ^{1}H NMR (500 MHz, DMSO-d_6) δ 4.00 (dq, *J* = 13.2, 6.9 Hz, 1H), 3.18 (p, *J* = 6.4 Hz, 1H), 2.45 (dd, *J* = 7.2, 6.1 Hz, 2H), 2.37 – 2.30 (m, 2H), 1.24 (d, *J* = 6.7 Hz, 4H), 1.17 – 1.08 (m, 8H).

3-(5,6-dimethyl-1H-benzimidazol-2-yl)-N,N-di(propan-2-yl)propenamide (MSU-43065). A 100 mL 24/40 round bottom flask was charged with 4,5-dimethylbenzene-1,2-diamine (0.299 g, 2.20 mmol), anhydrous 1,4-dioxane (6.00 mL). and 3-[bis(propan-2-yl)carbamoyl]propanoic acid (0.482 g, 2.40 mmol), followed by polyphosphoric acid (1.63 g, 16.0 mmol). The reaction vessel was heated to 105 °C under argon and stirred for 20 hours. The reaction was quenched by adding water (30.0 mL) and sodium carbonate (12.3 g, 117 mmol). The reaction mixture was partitioned with ethyl acetate and concentrated *in vacuo*. The crude material was purified by Reverse-phase Medium Pressure Liquid Chromatography (50 g C18 column, methanol in 25.0 mmolar ammonium formate). The resulting formate salt was then partitioned between ethyl acetate and a saturated sodium bicarbonate solution, the organic layer washed with brine, dried over sodium sulfate, filtered, and concentrated *in vacuo* to yield pure product as a powder (0.151 g, 22%). ^{1}H

NMR (500 MHz, DMSO-d_6) δ 11.88 (s, 1H), 7.20 (s, 2H), 4.11 – 3.95 (m, 1H), 3.46 (s, 1H), 2.94 (dd, J = 9.0, 6.3 Hz, 2H), 2.78 (dd, J = 9.0, 6.3 Hz, 2H), 2.26 (s, 6H), 1.27 (d, J = 6.7 Hz, 6H), 1.18 – 1.10 (m, 6H). APCI [M+H] calc'd = 302.2226 observed = 302.2274. m.p. = 182 ºC.

Scheme 3. Preparation of MSU-43085

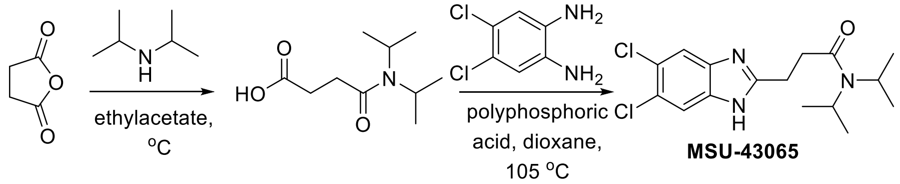

3-(5,6-dichloro-1H-1,3-benzodiazol-2-yl)-N,N-bis(propan-2-yl)propenamide (MSU-43085). A 100 mL 24/40 round bottom flask was charged with 4,5-dichlorobenzene-1,2-diamine (0.299 g, 2.20 mmol) and anhydrous 1,4-dioxane (6.00 mL). 3-[bis(propan-2-yl)carbamoyl]propanoic acid (0.482 g, 2.40 mmol) was added, followed by polyphosphoric acid (1.63 g, 16.0 mmol). The reaction vessel was heated to 105 ºC and stirred under an argon atmosphere for 20 hours. The reaction was quenched by adding water (30.0 mL) and sodium carbonate (12.3 g, 117 mmol). The reaction mixture was partitioned with ethyl acetate and concentrated *in vacuo*. The crude material was purified by Reverse-phase Medium Pressure Liquid Chromatography (50 g C18 column, methanol in 25.0 mmolar ammonium formate). The resulting formate salt was then partitioned between ethyl acetate and a saturated sodium bicarbonate solution, the organic layer washed with brine, dried over sodium sulfate, filtered, and concentrated *in vacuo* to yield pure product as a powder (0.151 g, 22%). ^{1}H NMR (500 MHz, DMSO-d6) δ 12.49 (s, 1H), 7.72 (s, 2H), 4.04 (hept, J = 6.8 Hz, 1H), 3.50 (s, 1H), 3.00 (dd, J = 8.5, 6.4 Hz, 2H), 2.82 (dd, J = 8.5, 6.4 Hz, 2H), 1.25 (d, J = 6.7 Hz, 6H), 1.13 (d, J = 6.6 Hz, 6H). APCI [M+H] calc'd = 342.1134 observed = 342.1168.

Scheme 4. Preparation of MSU-43165.

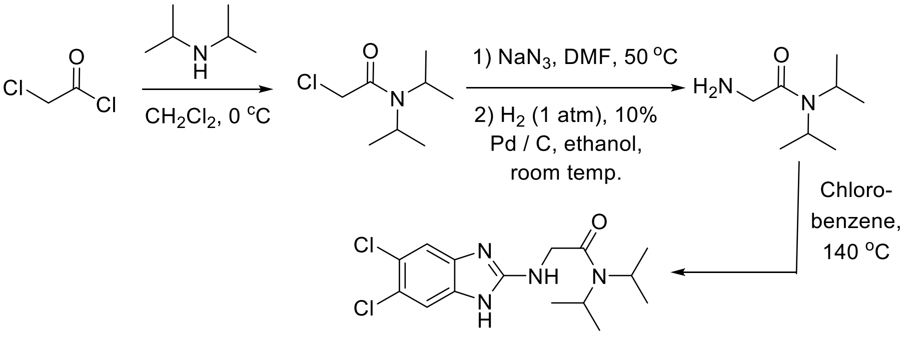

2-amino-N,N-bis(propan-2-yl)acetamide. 2-chloro-N,N-bis(propan-2-yl)acetamide (1.77 g, 10.0 mmol) was prepared according to the method described above, dissolved in dimethylformamide (20.0 mL), combined with sodium azide (1.95 g, 30.0 mmol), and heated to 50 ºC under argon for 19 hours. The reaction mixture was cooled to room temperature, partitioned between ethyl acetate and water, washed with brine, 1.00 N aqueous hydrochloric acid, brine, dried over sodium sulfate, filtered and concentrated to yield the product as an oil (1.82 yield, 99 %). ^{1}H

NMR (500 MHz, Chloroform-d) δ 3.87 (s, 2H), 3.72 (h, J = 6.7 Hz, 1H), 3.53 (s, 1H), 1.41 (d, J = 6.8 Hz, 6H), 1.28 – 1.18 (m, 6H). Spectra match that reported for this compound in *Eur. J. Med. Chem.*, **2013**, *69*, 338. This material was dissolved in ethanol (30.0 mL), stirred with 10 % palladium on carbon (0.304 g, 2.85 mmol). The reaction vessel was sealed and hydrogenated using a balloon filled with hydrogen for 14 hours. The reaction was filtered through Celite (washing with ethanol) and concentrated *in vacuo* to yield 2-amino-N,N-bis(propan-2-yl)acetamide as an oil (1.50 g, 95 %). ^{1}H NMR (500 MHz, Chloroform-d) δ 3.79 (dt, J = 13.4, 6.7 Hz, 1H), 3.42 (d, J = 18.0 Hz, 1H), 3.36 (s, 2H), 1.36 (d, J = 6.8 Hz, 6H), 1.15 (d, J = 6.6 Hz, 6H). Spectra match that reported in Monaghan, Sandra Marina; Mantell, Simon John, WO2000023457. 2-Amino-N,N-bis(propan-2-yl)acetamide (0.158 g, 1.00 mmol) was then added to a 100 mL 24/40 round bottom flask followed by a stir bar and 2,5,6-Trichloro-1H-benzo[d]imidazole (0.110 g, 0.500 mmol). The reaction vessel was sealed with a septum, flushed with argon, and chlorobenzene (5.00 mL) was added by syringe. The reaction mixture was heated to 140 ^{0}C for 20 hours, at which time it was cooled to room temperature and concentrated *in vacuo* to yield the crude product as a solid. This material was dissolved in methanol and purified by reverse-phase Medium Pressure Liquid Chromatography (415 g C_{18} column in a 0 to 100 % gradient of methanol/25 mM aqueous ammonium formate buffer). Fractions containing the product were combined and concentrated then taken up in ethyl acetate, washed with saturated aqueous sodium bicarbonate, brine, dried over sodium sulfate, filtered, and concentrated again. The material was finally purified by recrystallization from dichloromethane and hexanes to yield the pure product as a white solid (0.122 g, 71 % yield). ^{1}H NMR (500 MHz, DMSO-d_6) δ 10.86 (s, 1H), 7.32 (d, J = 36.8 Hz, 2H), 6.85 (s, 1H), 4.11 (s, 2H), 3.91 (s, 1H), 3.50 (s, 1H), 1.30 (d, J = 6.7 Hz, 6H), 1.16 (d, J = 6.6 Hz, 6H). ESI (+) calc'd for [M+Na] = 365.0911, found = 365.0917.

Scheme 5. Preparation of MSU-43165

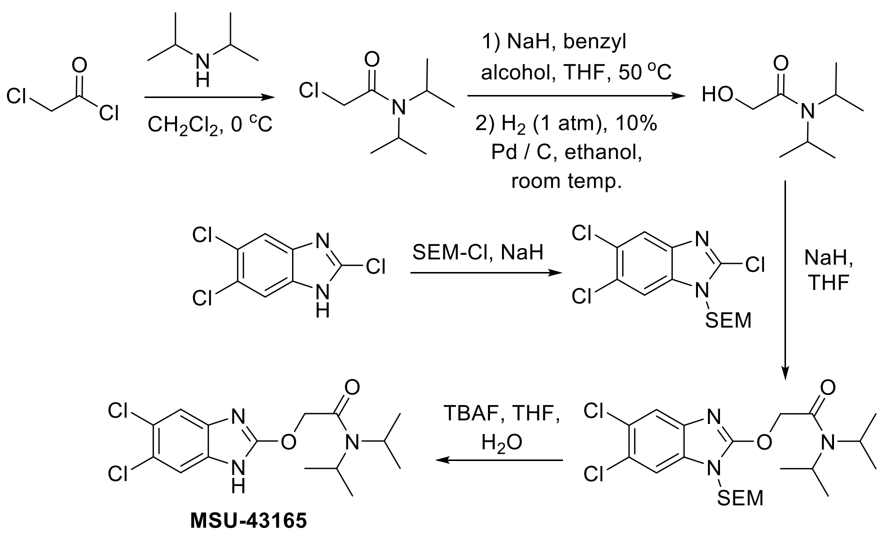

2-hydroxy-N,N-bis(propan-2-yl)acetamide. 2-chloro-N,N-bis(propan-2-yl)acetamide (2.27 g, 12.7 mmol) was dissolved in tetrahydrofuran (40.0 mL) and benzyl alcohol (1.62 g, 15.0 mmol). The reaction mixture was cooled to 0 ºC under argon and sodium hydride was added (0.537 g, 16.0 mmol). The reaction mixture was heated to 50 ºC for 19 hours, at which time it was quenched with a solution of saturated aqueous ammonium chloride (20.0 mL) and partitioned with ethyl acetate. The aqueous layer was extracted with ethyl acetate, the organic layers were combined, washed with brine, dried over sodium sulfate, filtered, and concentrated *in vacuo* to yield the crude product as an oil. The product was purified by silica gel chromatography (1:4 ethyl acetate in hexanes) to yield the pure product as an oil (2.89 g, 91 %, Miyatake, Tsuneo; Tanaka, Shigeyuki; Shimada, Atsuo, JP49011412 B.). ^{1}H NMR (500 MHz, Chloroform-d) δ 7.41 – 7.24 (m, 5H), 4.60 (s, 2H), 4.10 (s, 2H), 4.03 – 3.92 (m, 1H), 3.48 – 3.35 (m, 1H), 1.42 (d, J = 6.8 Hz, 6H), 1.17 (dd, J = 11.0, 6.4 Hz, 6H). This material was dissolved in ethanol (30.0 mL) and stirred with 10 % palladium on carbon (0.442 g, 4.15 mmol) under a hydrogen atmosphere applied with a balloon for 20 hours. The reaction mixture was filtered through celite (washing with ethanol) and concentrated *in vacuo* to yield the product as a white solid (1.59 g, 91 %, Scardovi, N.; Casalini, A.; Peri, F.; Righi, P., *Org. Lett.*, **2002**, *4*, 965.). 1H NMR (500 MHz, Chloroform-d) δ 4.07 (d, J = 4.2 Hz, 2H), 3.60 (dt, J = 13.3, 6.6 Hz, 2H), 3.48 (p, J = 6.7 Hz, 1H), 1.41 (d, J = 6.7 Hz, 6H), 1.19 (d, J = 6.5 Hz, 6H).

2,5,6-trichloro-1-{[2-(trimethylsilyl)ethoxy]methyl}-1H-1,3-benzodiazole. The title compound was prepared according to the general method described in Duane Burnett, Wen-Lian Wu, Thavalakulamgara Sasikumar, William Greenlee, Mary Caplen, Tao Guo, Rachael Hunter, US20050054628. A 100 mL 24/40 roundbottom flask was charged with 2,5,6-trichloro-1H-1,3-benzodiazole (0.424 g, 1.90 mmol) and dimethylformamide (10.0 mL). Under an argon atmosphere, sodium hydride was added (0.100, g, 3.0 mmol), and once gas evolution was complete, 2-(chloromethoxy)ethyl trimethylsilane (0.416 g, 2.50 mmol) was added via syringe. The mixture was stirred for 19 hours at which time water was added (20.0 mL). The mixture was extracted with ethyl acetate, the organic layer washed with brine, dried over sodium sulfate, decanted and concentrated *in vacuo* to yield the crude product which was purified by silica gel chromatography (3:7 ethyl acetate/hexanes) to yield the product (0.510 g, 76 %). ^{1}H NMR (500 MHz, DMSO-d_6) δ 8.12 (s, 1H), 7.95 (s, 1H), 5.64 (s, 2H), 3.54 (t, J = 7.9 Hz, 2H), 0.81 (t, J = 7.9 Hz, 2H), -0.12 (s, 9H).

2-[(5,6-dichloro-1-{[2-(trimethylsilyl)ethoxy]methyl}-1H-1,3-benzodiazol-2-yl)oxy]-N,N-bis(propan-2-yl)acetamide. A 50 mL 24/40 round bottom flask was charged with 2-hydroxy-N,N-bis(propan-2-yl)acetamide (0.318 g, 2.00 mmol) and tetrahydrofuran (5.00 ml). The mixture was cooled to 0 ºC, under an argon atmosphere, and sodium hydride added (0.100 g, 3.00 mmol). Once gas evolution stopped, 2,5,6-trichloro-1-{[2-(trimethylsilyl)ethoxy]methyl}-1H-1,3-benzodiazole (0.476 g, 1.30 mmol) was added as a solution in tetrahydrofuran (5.00 mL). The reaction was warmed to room temperature and stirred for 19 hours, at which time an aqueous solution of saturated ammonium chloride (20.0 mL) was added. The reaction was extracted with

ethyl acetate and the organic layer washed with brine, dried over sodium sulfate, decanted, and concentrated *in vacuo* to yield the crude product. This material was purified by silica gel chromatography (1:1 ethyl acetate/hexanes) to yield the final product as a white solid (0.510 g, 83 %). 1H NMR (500 MHz, DMSO-d6) δ 7.75 (s, 1H), 7.64 (s, 1H), 5.44 (s, 2H), 5.22 (s, 2H), 3.83 (p, J = 6.6 Hz, 1H), 3.64 – 3.56 (m, 2H), 3.48 (tt, J = 13.4, 7.3 Hz, 1H), 1.24 (d, J = 6.7 Hz, 6H), 1.17 (d, J = 6.4 Hz, 6H), 0.88 – 0.78 (m, 2H), -0.09 (s, 9H).

2-[(5,6-dichloro-1H-1,3-benzodiazol-2-yl)oxy]-N,N-bis(propan-2-yl)acetamide (MSU-43170). 2-[(5,6-dichloro-1-{[2-(trimethylsilyl)ethoxy]methyl}-1H-1,3-benzodiazol-2-yl)oxy]-N,N-bis(propan-2-yl)acetamide was dissolved in tetrahydrofuran (10.0 mL) under an argon atmosphere and tetrabutylammonium fluoride was added as a 1.00 M solution in tetrahydrofuran (10.0 mL), followed by 0.050 mL of water. The reaction mixture was heated to 50 ºC for 70 hours at which time it was concentrated *in vacuo*. The crude material was purified by Reverse-phase Medium Pressure Liquid Chromatography (50 g C18 column, methanol in 25.0 mmolar ammonium formate). The product was partitioned between ethyl acetate and saturated sodium bicarbonate. The organic layer was then washed with brine, dried over sodium sulfate, filtered, and concentrated *in vacuo*. The product was additionally purified by silica gel chromatography (15:85 methanol/dichloromethane) to yield an oil. This material was recrystallized from dichloromethane/hexanes to yield the product as white needles (0.092 g, 26 %). ^{1}H NMR (500 MHz, DMSO-d_6) δ 12.37 (s, 1H), 7.71 – 7.38 (m, 2H), 5.14 (s, 2H), 3.83 (p, *J* = 6.7 Hz, 1H), 3.50 (dt, *J* = 13.9, 4.8 Hz, 1H), 1.23 (dd, *J* = 39.5, 6.6 Hz, 12H). HRMS ESI (+) calc'd for [M+H] = 344.0928, 344.0941.
